# Association of distance between hospitals and volume of shared admissions

**DOI:** 10.1186/s12913-022-08931-1

**Published:** 2022-12-15

**Authors:** Sara D. Turbow, Teg Uppal, Howard H. Chang, Mohammed K. Ali

**Affiliations:** 1 Department of Medicine, Division of General Internal Medicine, School of Medicine, Emory University, 49 Jesse Hill Jr Dr SE, Atlanta, GA 30303 USA; 2 Department of Family and Preventive Medicine, School of Medicine, Emory University, Atlanta, GA USA; 3 Hubert Department of Global Health, Rollins School of Public Health, Emory University, Atlanta, GA USA; 4 Department of Biostatistics and Bioinformatics, Rollins School of Public Health, Emory University, Atlanta, USA

**Keywords:** Readmission, Care fragmentation, Health information exchange, Healthcare market

## Abstract

**Background:**

To assess whether decreasing distance between hospitals was associated with the number of shared patients (patients with an admission to one hospital and a readmission to another).

**Methods:**

Data were from the Healthcare Cost and Utilization Project’s State Inpatient Databases (Florida, Georgia, Maryland, Utah [2017], New York, Vermont [2016]) and the American Hospital Association Annual Survey (2016 & 2017). This was a cross-sectional analysis of patients who had an index admission and subsequent readmission at different hospitals within the same year. We used unadjusted and adjusted linear regression to evaluate the association between the number of shared patients and the distance between admission-readmission hospital pairs.

**Results:**

There were 691 hospitals in the sample (247 in Florida, 151 in Georgia, 50 in Maryland, 172 in New York, 58 in Utah, and 13 in Vermont), accounting for a total of 596,772 admission-readmission pairs. 32.6% of the admission-readmission pairs were shared between two hospitals. On average, a one-mile decrease in distance between two hospitals was associated with of 3.05 (95% CI, 3.02, 3.07) more shared admissions. However, variability between states was wide, with Utah having 0.37 (95% CI 0.35, 0.39) more shared admissions between hospitals per one-mile shorter distance, and Maryland having 4.98 (95% CI 4.87, 5.08) more.

**Conclusions:**

We found that proximity between hospitals is associated with higher volumes of shared admissions.

**Supplementary Information:**

The online version contains supplementary material available at 10.1186/s12913-022-08931-1.

## Introduction

Interhospital care fragmentation, when a patient has a readmission to a different hospital than they were previously discharged from, has negative impacts on both patients and the health system, including higher rates of in-hospital mortality [1–5], longer lengths-of-stay [1, 2], higher costs per readmission [6–8], as well as increased risk of subsequent readmissions [9–11]. Previous work has shown that up to 25% of readmissions are fragmented [7], but these readmissions are likely not distributed evenly across patients and hospitals. Understanding what patient characteristics, clinical situations, and hospital or healthcare system structures increase the likelihood of a fragmented readmission is essential to minimize fragmented care where possible, and where to focus care coordination resources to mitigate the negative effects of fragmented readmissions when they do occur.

Patient-, clinical-, and hospital/healthcare system characteristics may all affect the likelihood of a fragmented readmission. If a patient is not satisfied with the care they received in the past, they might intentionally seek care elsewhere [12]. They may also receive care at the closest hospital, out of necessity or familiarity [12–17]—even if their previous care occurred elsewhere. A previous study examining patients with fragmented readmissions following an initial admission for major surgery found that patients whose readmissions were fragmented lived, on average, farther away from the admission hospital than did patients who were readmitted to the same hospital [4]. The patient’s diagnosis, their need for specialist care, or a patient’s illness severity may affect the odds of a fragmented readmission. Healthcare-system level characteristics, including hospital availability, hospital bed availability, insurance coverage, and mode of transport to the hospital (i.e., taking an ambulance versus a personal vehicle) may all affect the likelihood that a patient’s readmission is to a different hospital [18, 19].

Few studies have considered fragmented care from the perspective of a hospital rather than that of a patient. Each fragmented readmission to the hospital represents another hospital to coordinate care and share information with. When fragmentation is examined across a geographic area such as a city or state, a hospital could be responsible for sharing patients with dozens of other hospitals [20]: one recent study investigating dispersion of patients across networks of hospitals found that hospitals shared Medicare patients with a median of 31 other hospitals [21].

To further our understanding of fragmented care from the hospital, rather than the patient, viewpoint, we sought to determine if there was an association between the distance between hospitals and the number of admission-readmission pairs they share (fragmented readmissions), and how hospital, patient, and market-level factors impact the number of shared admission-readmission pairs.

## Methods

In order to estimate the association between distance and the volume of shared admission-readmission pairs between hospitals, i.e., an initial or index admission to one hospital and then a readmission to a different hospital, we conducted a cross-sectional analysis of six geographically diverse Agency for Healthcare Research and Quality Healthcare Cost and Utilization Project (HCUP) State Inpatient Databases (SID): Florida, Maryland, Utah, and Georgia from 2017 and New York and Vermont from 2016. Each SID contains all nonfederal hospital discharges from a single year in a single state [22]. These states were chosen because they included linkage variables that allow tracking of an individual patient’s hospital admissions over the course of the year and allowed for linkage to American Hospital Association (AHA) data. The SID is available to the public at https://www.hcup-us.ahrq.gov/sidoverview.jsp.

AHA Annual Surveys [23] from 2016 and 2017 were used to obtain data regarding hospital characteristics such as size, teaching status, and ownership, as well as location.

### Variable definitions

Inpatient admissions of patients 18 years of age and older and any readmissions in the calendar year were included in the analysis. Interhospital transfers were excluded; interhospital transfers usually occur when a patient needs a higher level of care and are generally planned and given more support than a fragmented readmission. The SID denotes admissions resulting from transfers via the variable “TRAN_IN.” If “TRAN_IN” was coded as a transfer from another acute care hospital, the observation was removed. We created admission-readmission pairs for patients; if a patient had multiple readmissions, multiple pairs were created. For example, if a patient was admitted on day 1 (admission #1), readmitted on day 20 (admission #2), and then readmitted again on day 40 (admission #3), two pairs would be created: admissions 1 + 2 and admissions 2 + 3. Admissions and readmissions could be for any reason and did not need to have related or similar diagnoses. A readmission was determined to be at a different hospital than the prior admission if the two hospitals had different AHA identification numbers.

The primary predictor of interest was the distance between the hospitals in the admission-readmission pair. Euclidean distance between hospitals was measured using the hospital latitude and longitude provided in the AHA Annual Survey. The primary outcome was the absolute number of shared admission-readmission pairs between two hospitals. When counting the number of shared admissions, no directionality was applied, i.e., an admission to hospital A and subsequent readmission to hospital B was counted the same as an admission to hospital B and readmission to hospital A.

Hospital characteristics and market conditions were included in adjusted models. These were included because both hospital characteristics and hospital availability may impact the prevalence of care fragmentation and the likelihood of shared patient admissions between hospitals. The hospital characteristics of interest included: teaching status, type of hospital, size (number of beds), ownership, rural/urban status, and area racial/ethnic makeup. A hospital was considered to be a teaching hospital if the hospital answered that it had programs accredited by the American Council of Graduate Medical Education, the American Osteopathic Association, the Council of Teaching Hospitals, or if it was affiliated with a medical school [23]. Hospitals were divided into two types: general medical/surgical and “other,” which included specialty and pediatric hospitals. Hospital ownership was grouped into government, church, other not-for-profit, and for-profit. Urban/rural status was identified by the Rural Urban Commuting Area Codes of the hospital [24], and was divided into metropolitan, micropolitan, and rural areas. Finally, racial/ethnic makeup was measured as the percent of patients who identified as white in the patient’s zip code tabulation area (ZCTA). ZCTAs are geographic areas created by the U.S. Census that approximate zip codes for more accurate statistical analysis [25]. ZCTAs were obtained from the American Community Survey and were linked to the data via zip code matching where 5-digit zip codes were available; where 3-digit zip codes were available the data was matched on the first 3 characters of the zip code [26].

Each of the hospital characteristics was obtained for both the admission and readmission hospital and then categorized as a characteristic of the admission-readmission pair. For example, if the admission hospital was a teaching hospital but the readmission hospital was not, the admission-readmission pair category would be “teaching-nonteaching.” These pairs were directional, i.e. an index admission to a teaching hospital and readmission to a nonteaching hospital would not be coded the same as an admission to a nonteaching hospital and readmission to a teaching hospital. The admission-readmission pair categories were used as covariates in the regression models.

Market characteristics of the geographic area were measured via the Herfindahl–Hirschman Index (HHI) [27]. The HHI is a common method used in business and research settings to measure market concentration [28]; values range from 0–10,000, with higher numbers indicating more market concentration among a smaller number of hospitals. This is of particular importance to this analysis because the number of shared patients between hospitals may be directly associated with how many other hospitals are in the “market.” First, the “market” was defined as the radius around a hospital that contained 75% of its admissions. This was done by measuring the distance between each patient’s zip code reported in the SID and the hospital zip code in the AHA Annual Survey using the SAS “zipcitydistance” function. Then, the other hospitals within that radius were identified by measuring the distance between hospitals. Next, the percent of total discharges in the market belonging to each hospital in the market was measured. Finally, the HHI was calculated by summing the squares of the fraction of discharges of each hospital in the market [18].

### Statistical analysis

We first identified all admission-readmission pairs that occurred in the same hospital and removed them. This analysis was limited to admission-readmission pairs in which the admission and readmission occurred in different hospitals. To determine if differences existed across states, we performed univariate statistics to describe the states in the sample, then examined each state individually. Hospital characteristics for admission hospitals and readmission hospitals between states were compared separately using ANOVA and t-tests. We then estimated unadjusted and adjusted linear regression models to assess the relationship of distance between two hospitals and their volume of shared admission-readmission pairs. For the adjusted models, the characteristics of the admission-readmission pair were first included as covariates (admission hospital bed size/readmission hospital bed size, admission hospital teaching status/readmission hospital teaching status, admission hospital ownership/readmission hospital ownership, admission hospital type/readmission hospital type, admission hospital rural/urban status/readmission hospital rural/urban status). A model including admission-readmission pair and patient characteristics (admission-readmission pair characteristics and percent of patients who identified as white in the ZCTA) was then created, followed by one adjusting for only market characteristics, followed by a fully adjusted model. Each of these models was created for an individual state and for all states together. For the multi-state analyses, dummy variables for each state were included.

We performed several sensitivity analyses. First, to test whether the number of patients two hospitals share was impacted by whether the hospitals were members of the same hospital/healthcare system, we stratified the analysis by “same system” and “different system” status of the admission-readmission hospital pair. Next, to determine the impact of other hospital characteristics, we included trauma hospital status, critical access hospital designation, whether the hospital identified as part of an accountable care organization (ACO), and whether the hospital offered Medicare Advantage, Medicaid Managed Care, marketplace insurances, small or large group insurance, or another type of insurance. A sensitivity analysis limited to 30-day readmissions was also performed. Additionally, because patients with multiple readmissions could carry undue weight in this analysis, a sensitivity analysis was performed in which only the first admission-readmission pair was included for each patient. Finally, to test if the association between distance between hospitals and number of shared patients was sensitive to the volume of admissions at the admission and readmission hospitals, we performed a sensitivity analysis where the outcome was the proportion of shared admissions over the total volume of admissions at the index and readmission hospitals. All statistical analyses were completed in SAS 9.4 (Cary, NC).

This study was deemed exempt by the Emory Institutional Review Board. Table cells with values ≤ 11 were suppressed to comply with the HCUP data use agreement.

## Results

There were 691 hospitals in the sample: 247 in Florida, 151 in Georgia, 50 in Maryland, 172 in New York, 58 in Utah, and 13 in Vermont (Table 1). Florida had 2,425,843 discharges in the original dataset, after applying exclusion criteria and transforming the unit of observation into fragmented admission-readmission pairs for an individual patient, the final sample size for Florida was 278,345 (34.5% of all admission-readmission pairs were fragmented). Georgia began with 1,088,905 discharges, 84,265 (32.5% of all admission-readmission pairs) pairs remained in the final sample. Maryland went from 608,363 discharges to 54,156 pairs (36.0% of all admission-readmission pairs), New York 2,293,306 discharges to 164,501 pairs (28.8% of all admission-readmission pairs), Utah 225,744 discharges to 14,523 pairs (32.4% of all admission-readmission pairs), and Vermont 45,047 discharges to 1,182 pairs (11.0% of all admission-readmission pairs). The sample size for all states pooled together was 596,772 admission-readmission pairs. Overall, 32.6% of admission-readmission pairs were shared between two different hospitals. Full details of the sample development can be seen in Additional file 1: Appendix 1.

**Table 1:** Characteristics of hospitals in the sample

Variable		All States (*N* = 691) (95% CI)	Florida (*N* = 247)	Georgia (*N* = 151)	Maryland (*N* = 50)	New York (*N* = 172)	Utah (*N* = 58)	Vermont (*N* = 13)	p
**Bedsize**	**< 100 Beds**	37.3% (33.7–40.9)	38.9%	47.7%	22.0%	20.9%	56.9%	76.9%	0.704
	**100–199 Beds**	24.0% (20.8–27.2)	22.7%	24.5%	28.0%	25.0%	25.9%	–^d^	
	**200–299 Beds**	15.2% (12.5–17.9)	15.0%	9.3%	24.0%	20.9%	8.6%	–^d^	
	**300–399 Beds**	8.1% (6.2–10.1)	9.7%	6.6%	12.0%	7.6%	5.2%	0	
	**400–499 Beds**	5.5% (3.8–7.2)	4.7%	4.0%	10.0%	8.%	0	–^d^	
	**≥ 500 Beds**	9.8% (7.6–12.1)	8.9%	8.0%	4.0%	17.4%	3.5%	0	
**Teaching**	**Non-Teaching**	57.0% (53.3–60.7)	59.9%	72.2%	40.0%	37.2%	72.4%	84.6%	0.027
	**Teaching**	43.0% (39.3–46.7)	40.1%	27.8%	60.0%	62.8%	27.6%	15.4%	
**Ownership**	**Government**	13.3% (10.8–15.9)	9.3%	23.8%	0	14.0%	15.5%	0	< 0.001
	**Church**	6.5% (4.7–8.4)	6.1%	5.3%	12.0%	9.3%	0	0	
	**Other Not-for-profit**	53.1% (49.4–56.8)	32.4%	49.0%	86.0%	76.7%	43.1%	100%	
	**For Profit**	27.1% (23.8–30.3)	52.2%	21.9%	2.0%	0	41.4%	0	
**Hospital Type**	**General Medical/Surgical**	82.5% (79.7–85.3)	72.5%	83.4%	92.0%	94.2%	75.9%	100%	< 0.001
	**Other**	17.5% (14.7–20.3)	27.5%	16.6%	8.0%	5.8%	24.1%	0	
**Urban/Rural**	**Metropolitan**	79.0% (76.0–82.1)	91.5%	63.4%	92.0%	79.1%	69.0%	15.6%	< 0.001
	**Micropolitan**	9.7% (7.5–11.9)	63.6%	14.6%	4.0%	13.4%	8.6%	38.5%	
	**Rural**	11.3% (8.9–13.7)	4.5%	21.9%	4.0%	7.6%	22.4%	46.2%	
**Herfindahl–Hirschman Index 75% (mean, SD)**^**a**^		3407.2 (± 2568.0)	3505.1 (± 2354.4)	3639.2 (± 2902.2)	3207.7 (± 2892.5)	3160.0 (± 2482.6)	3353.1 (± 3366.4)	8056.2 (± 2604.9)	< 0.001
**Distance between Hospitals (mean, SD)**^**b**^		33.1 (± 55.2)	34.7 (± 61.4)	48.9 (± 62.7)	22.2 (± 27.3)	24.3 (± 38.6)	48.3 (± 84.6)	61.7 (± 27.7)	< 0.001
**Number of Shared Patients (Mean, SD)**^**c**^		439.3 (± 616.5)	573.3 (± 789.1)	289.3 (± 327.9)	337.6 (± 361.4)	353.1 (± 404.9)	120.1 (± 120.3)	135.6 (± 84.1)	< 0.001
**Number of Shared Patients per 10,000 Admission-Readmission Pairs**		–	20.6	34.3	62.2	21.5	82.6	1142.1	< 0.001

^a^Herfindahl–Hirschman Index is a measure of market saturation, here it is based on the market/geographic area from which 75% of an individual hospitals admissions come from

^b^Distance between hospitals was measured between the latitude and longitude of the hospital as reported in the AHA Annual Survey

^c^The number of shared patients is the number of patients who have an admission and readmission between a specific hospital pair. This is a nondirectional measure

^d^Data suppressed

Table 1 displays the descriptive characteristics of the hospitals in the sample, by state and pooled across all states. Briefly, there was no difference among distribution of hospital sizes across states (*p* = 0.70). Across all states, 43.0% of hospitals were teaching hospitals. Hospitals in Georgia (27.8%), Utah (27.6%), and Vermont (15.4%) were less likely to be teaching hospitals than those in other states (*p* = 0.03). 27.1% of hospitals across the sample were for-profit; a higher proportion of hospitals were for-profit in Florida (52.2%) and Utah (41.4%) than in other states (*p* < 0.001). Overall, 11.3% of hospitals in the sample were in rural areas. Georgia (21.9%), Utah (22.4%), and Vermont (46.2%) had higher proportions of rural hospitals than other states (*p* < 0.001). The mean HHI was 3407.2, and Vermont had the highest mean HHI/greatest market concentration of any state (8056.2) and the greatest average distance between hospitals (61.7 miles). The mean distance between hospitals across all states was 33.1 miles.

In absolute numbers, Florida had the highest mean number of shared admissions between hospitals over the course of a year (*n* = 573.3) and Utah the lowest (*n* = 120.1); the range across all hospital pairs was 1–4,848. When the rates were standardized per 10,000 fragmented admission-readmission dyads, Vermont had the highest number with 1,142.1 shared patients per 10,000 fragmented admissions and Florida the lowest, with 20.6 shared patients between hospitals per 10,000 fragmented admissions.

The distribution of hospital characteristics of admission hospitals in admission-readmission pairs is shown in Additional file 1: Appendix 2 and the distribution of hospital characteristics for the readmission hospitals is shown in Additional file 1: Appendix 3. Across all states, a higher proportion of readmissions were to “other” type hospitals than index admissions (6.4% of index admissions v. 11.0% of readmissions). Distributions of other characteristics between index and readmission hospitals was similar. Utah and Vermont had the highest percent of white patients per admission hospital (87.1 and 95.7%, respectively).

Table 2 shows the results of the unadjusted and adjusted linear regression models. In the pooled, unadjusted model, a one-mile increase in distance between hospitals was associated with, on average, 3.19 (95% CI, -3.22, -3.16) fewer shared admissions between them. This can be interpreted in the inverse direction as well: for every one-mile decrease in distance between two hospitals, there is an associated increase of 3.19 (95% CI 3.16, 3.22) patients that they share. Variability between states was wide, with Utah having 0.39 (95% CI 0.37, 0.45) more shared admissions per hospital per one-mile shorter inter-hospital distance, and Maryland having 5.00 (95% CI 4.89, 5.09) more. When we included the racial/ethnic make-up at the ZCTA level, all states except Vermont had a slight increase in the value of the regression coefficient. The regression coefficient for Vermont, conversely, decreased (0.66, 95% CI 0.57, 0.75). In fully adjusted models, the range across states was from 0.37–4.98 (95% CI 0.35–0.39, 5.08–4.87).

**Table 2:** Linear regression models, association between increasing distance between hospitals and volume of shared patients

Model	All^a^	Florida	Georgia	Maryland	New York	Utah	Vermont
Unadjusted	-3.19 (-3.22, -3.16)	- 3.46 (-3.51, -3.41)	-2.41 (-2.45, -2.39)	-5.00 (-5.09, -4.89)	-3.28 (-3.33, -3.23)	-0.39 (-0.45, -0.37)	-1.13 (-1.29, -0.97)
Hospital Characteristics	-2.87 (-2.90, -2.85)	-3.41 (-3.45, -3.37)	-2.26 (-2.30, -2.24)	-5.10 (-5.12, -4.92)	-2.88 (-2.93, -2.84)	-0.34 (-0.36, -0.32)	-1.24 (-1.38, -1.10)
Hospital + Patient Characteristics	-2.95 (-2.98, -2.93)	-3.46 (-3.50, -3.41)	-2.35 (-2.38, -2.32)	-4.92 (-5.02, -4.82)	-3.00 (03.05, -2.95)	-0.37 (-0.39, -0.34)	-0.66 (-0.75, -0.57)
Market	-3.16 (-3.19, -3.14)	-3.50 (-3.55, -3.45)	-2.37 (-2.40, -2.34)	-4.81 (-4.92, -4.70)	-3.20 (-3.25, -3.15)	-0.31 (-0.34, -0.29)	-1.15 (-1.30, -1.00)
All	-3.05 (-3.07, -3.02)	-3.52 (-3.56, -3.47)	-2.36 (-2.39, -2.33)	-4.98 (-5.08, -4.87)	-3.11 (-3.16, -3.08)	-0.37 (-0.39, -0.35)	-0.63 (-0.72, -0.55)

Unadjusted: Modeling the relationship between index and readmission hospital and volume of shared patients

Hospital Characteristics: bed size, teaching status, hospital ownership, and type of hospital—note each covariate is the characteristics of the pair (ex. index-teaching, readmit-nonteaching)

Hospital and Patient Characteristics: Above plus percent of white patients in the zip code tabulation area of the patient’s home zip code

Market: HHI (75% of market)

All: Hospital, patient, market as above

^a^Dummy variables for states were used in the multi-state analysis

In our sensitivity analyses, we found similar results to what is described above (Additional file 1: Appendix 4–11.) We observed that same system admission-readmission pairs had a larger increase in the number of shared patients than did different system admission-readmission pairs. For example, New York had 13 more patients shared per one mile decrease in distance between same-system pairs (-13.11, 95% CI -13.50, -12.72) and only 4 more patients share per one mile decrease in distance between different hospitals in different systems (-4.04, 95% CI -4.13, -3.95). When the proportion of patients shared was the outcome, rather than the absolute count of shared admissions, the results were similar: in the pooled, fully-adjusted model, a one mile increase in distance was associated with -0.020% decrease in the proportion of shared admissions over the total number of admissions at the admission and readmission hospitals (95% CI -0.020, -0.019, Additional file 1: Appendix 11).

## Discussion

While the distance between a patient’s home and a hospital has been examined as a potential risk factor for fragmented readmissions [4], the association between the distance between two hospitals and the number of shared fragmented admissions between them has previously gone unstudied. As hospitals consolidate in metropolitan areas [29] and healthcare information technology infrastructure crawls towards interoperability [30], it is important for hospitals to identify where else their patients are receiving care in order to understand gaps in their information exchange and care coordination efforts, the impact this may have on patient outcomes, and potential solutions to these challenges. Across six diverse states, we found an association between closer proximity between hospitals and higher volume of shared admissions, although it is highly variable by state and influenced by other factors.

The large variability between states suggests that there remain unmeasured factors influencing the volume of shared admissions between hospitals; these could include policy differences, ambulance use, and patient choice, among others. In 2014, Maryland introduced the Readmissions Reduction Incentive Program (RRIP), with the goal of reducing the statewide readmission rate for Medicare fee-for-service beneficiaries to match the national readmission rate by 2019 [31]. Like the national CMS Hospital Readmission Reduction Program, readmissions still “count” if they occur at a different hospital than the index hospital. Policies such as these may be more likely to impact the incidence of readmissions overall, rather than motivating hospitals to prevent fragmented/different hospital readmissions. In a study of Canadian heart failure patients with fragmented readmissions, ambulance use was associated with a 20% greater odds of a readmission to a different hospital [19]. A patient’s desire to receive care at a different hospital than they were previously admitted to may also play a role: in previous work, we showed that patients who left their index admission against medical advice, i.e., patients who were likely dissatisfied with the care they received, were twice as likely to be readmitted at a different hospital than were patients who did not leave their index admission against medical advice [7]. Relatedly, receiving care at a different hospital may be beneficial to patients who require a higher level or specialty care. Further work should examine both patient reasons for seeking care at a different hospital and whether these fragmented readmissions are medically “appropriate” or “inappropriate.”

Notably, our regression results only measured volume of admissions/readmissions between a *pair* of hospitals. While the models showed a small increase in the number of shared admissions per mile decrease of distance between hospitals, each hospital is part of multiple pairs, leading to a likely larger effect of distance on fragmented readmissions across an entire healthcare system in a city or state [20]. As described in Table 1, the mean number of shared admissions between hospitals ranged from 120–573, and the maximum number of shared admissions between two hospitals was 4,848. For each hospital, which may have dozens or hundreds of shared admissions with several hospitals, both near and far, these data could support decisions about prioritizing information sharing, care coordination, ambulance triage, and patient education to reduce fragmented readmissions or mitigate its poor outcomes. From a business standpoint, this information may help hospitals understand who their competitors are in their market.

These results could also inform policies around HIE development and implementation. Our finding that the number of shared admissions is lower with greater distance between hospitals could support a focus on local/state HIE development over regional or national HIE. It is important to note that we did not measure hospital pairs that cross state lines in this analysis, and in some cases a hospital’s closest neighboring hospitals may be in different states. This is a gap in current information sharing networks that regional and national HIE are trying to address [32].

This study has several important limitations. First, this was a secondary data analysis, so we are limited by what is available in the data set. For example, not all states had variables for the month the admission or readmission occurred in, limiting our ability to assess seasonal changes in the observed results. Additionally, because this study is limited to a single year of data and data cannot be linked across years, patients whose index admission occurred later in the year have a lower chance of being readmitted in the same year. However, when we limited the analysis to 30-day readmissions, the results were similar. Similarly, admissions are limited to those that occur in an individual state, and cross-state linking is not possible. While the 6 states in our sample were geographically diverse, each state has a different health care ecosystem (as shown in the differences in the analyses of individual states) and results may vary when additional states are examined. Relatedly, we only have data on patients who were admitted, meaning that we cannot measure out-of-hospital outcomes that may have an impact on the number of shared patients between hospitals. For example, if a greater distance between hospitals is associated with higher rates of prehospital mortality, this could introduce bias into our results. Additionally, the AHA survey is voluntary and does not have a 100% response rate, which could lead to nonresponse bias in the sample.

As hospitals look for solutions to care fragmentation and the fragmented information sharing that travels along with it, the results of this study can help inform local strategy. Proximity matters, so efforts to create local information sharing networks may be effective. However, if a hospital is geographically close to hospitals outside of a traditional HIE network (i.e. across state lines), information sharing might still need to be pursued. Other local factors such as ambulance networks and insurance patterns could affect these results and should be examined as they may impact the number of fragmented readmissions shared between hospitals.

## Supplementary Information

**Additional file 1:** **Appendix1.** Sample development diagram. **Appendix 2.** Distribution of hospitalcharacteristics of admission hospitals in admission-readmission dyads. **Appendix 3.** Distribution of hospitalcharacteristics of readmission hospitals in dyads. **Appendix 4.** Sensitivity analyses for additional hospital/payer characteristics, all readmissions. **Appendix 5.** Distribution of hospital characteristics of index hospitals in dyads, 30-day readmissions. **Appendix 6.** Distribution of hospital characteristics of readmission hospitals in dyads, 30-day readmissions. **Appendix 7.** Linear regression models, 30-day readmissions. **Appendix 8.** Distribution of hospital characteristics of index hospitals in dyads, first admission-readmission pair only. **Appendix 9.** Distribution of hospital characteristics of readmission hospitals in dyads, first admission-readmission pair only. **Appendix 10.** Linear regression models, first admission-readmission pair only. **Appendix 11.** Linear regression models, outcome is percent of total admissions to admission and readmission hospital.

## Data Availability

The data that support the findings of this study are available from the Healthcare Cost and Utilization Project but restrictions apply to the availability of these data, which were used under license for the current study, and so are not publicly available. Data are however available from the authors upon reasonable request and with permission of the Healthcare Cost and Utilization Project.
