# Supplementary material for: Association of distance between hospitals and volume of shared admissions
Source: BMC Health Serv Res. 2022 Dec 15;22:1528. doi: 10.1186/s12913-022-08931-1 (PMC9753317; doi:10.1186/s12913-022-08931-1)
Supplement: Supplementary file 1 — Additional file 1: Appendix1. Sample development diagram. Appendix 2. Distribution of hospitalcharacteristics of admission hospitals in admission-readmission dyads. Appendix 3. Distribution of hospitalcharacteristics of readmission hospitals in dyads. Appendix 4. Sensitivity analyses for additional hospital/payer characteristics, all readmissions. Appendix 5. Distribution of hospital characteristics of index hospitals in dyads, 30-day readmissions. Appendix 6. Distribution of hospital characteristics of readmission hospitals in dyads, 30-day readmissions. Appendix 7. Linear regression models, 30-day readmissions. Appendix 8. Distribution of hospital characteristics of index hospitals in dyads, first admission-readmission pair only. Appendix 9. Distribution of hospital characteristics of readmission hospitals in dyads, first admission-readmission pair only. Appendix 10. Linear regression models, first admission-readmission pair only. Appendix 11. Linear regression models, outcome is percent of total admissions to admission and readmission hospital. [file 12913_2022_8931_MOESM1_ESM.docx]

Appendix 1: Sample Development Diagram


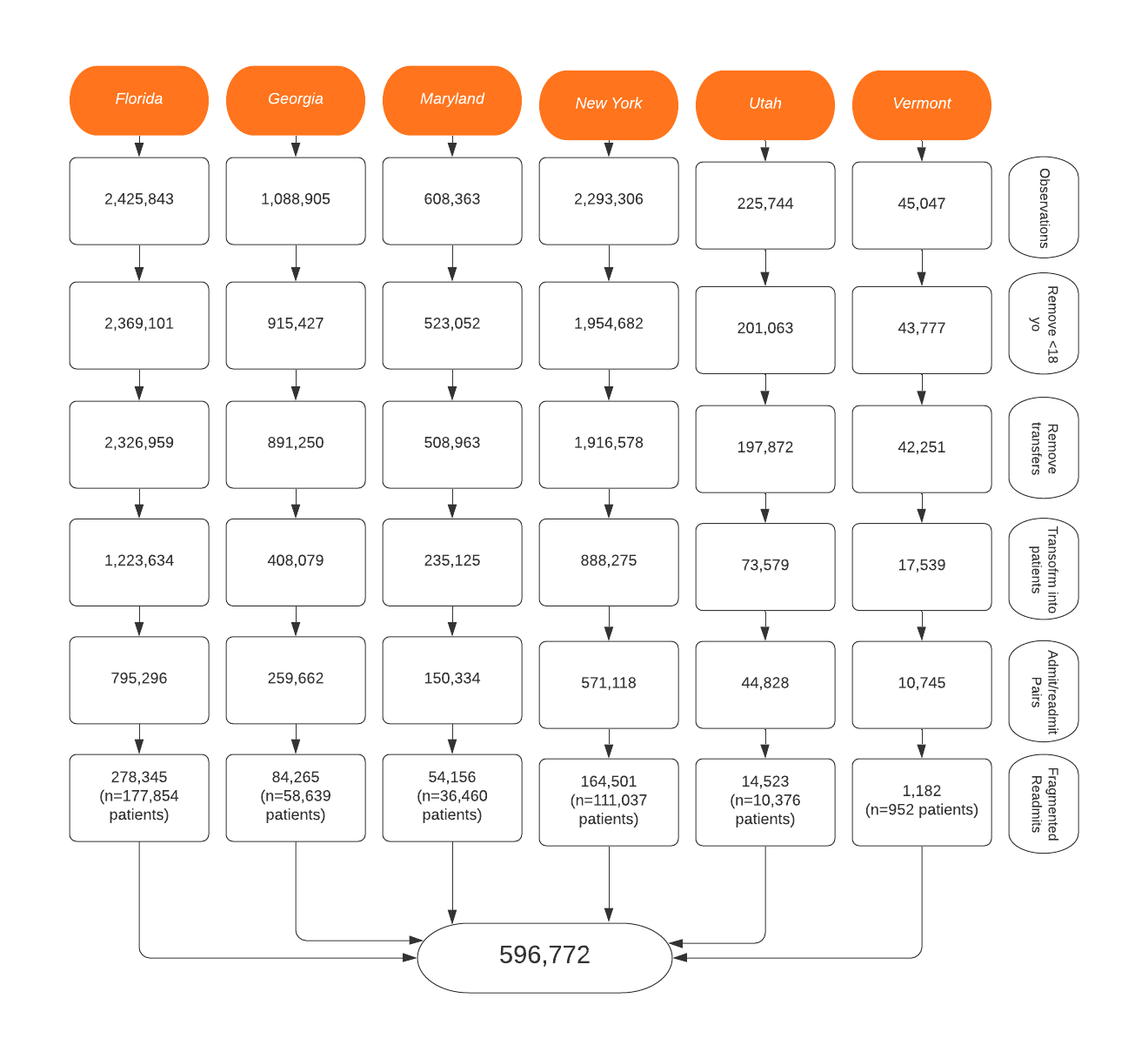


**Appendix 2: Distribution of Hospital Characteristics of Admission Hospitals in Admission-Readmission Dyads**

| **Variable** | | **All States**  **(n=596,772)**  **(%, 95% CI)** | **Florida (N=278,345)** | **Georgia (n=84,265)** | **Maryland (n=54,156)** | **New York (n=164,501)** | **Utah (n=14,323)** | **Vermont (n=1,182)** | **p** |
| --- | --- | --- | --- | --- | --- | --- | --- | --- | --- |
| **Bedsize** | **<100 Beds** | 8.9% (8.8-8.9) | 10.3% | 13.4% | 7.6% | 3.4% | 20.0% | 40.7% | <0.001 |
|  | **100-199 Beds** | 16.2% (16.1-16.3) | 18.2% | 15.5% | 19.7% | 11.2% | 27.2% | 9.8% |  |
|  | **200-299 Beds** | 18.2% (18.1-18.3) | 17.7% | 17.4% | 26.8% | 16.7% | 18.3% | 13.7% |  |
|  | **300-399 Beds** | 14.4% (14.3-14.5) | 16.8% | 11.4% | 17.5% | 11.0% | 13.7% | 0 |  |
|  | **400-499 Beds** | 10.9% (10.8-11.0) | 9.9% | 11.8% | 14.2% | 11.8% | 0 | 35.8% |  |
|  | **≥500 Beds** | 31.9% (31.3-31.6) | 27.2% | 30.5% | 14.2% | 46.0% | 20.9% | 0 |  |
| **Teaching** | **Non-Teaching** | 26.8% (26.7-26.9) | 34.7% | 37.0% | 15.6% | 10.7% | 37.2% | 50.5% | <0.001 |
|  | **Teaching** | 73.2% (73.1-73.4) | 65.3% | 63.1% | 84.4% | 89.3% | 62.8% | 49.5% |  |
| **Ownership** | **Government** | 12.4% (12.3-12.5) | 13.3% | 4.6% | 0 | 19.9% | 13.6% | 0 | <0.001 |
|  | **Church** | 6.6% (6.5-6.7) | 4.4% | 4.6% | 14.7% | 9.3% | 0 | 0 |  |
|  | **Other Not-for-profit** | 57.0% (56.9-57.1) | 38.3% | 73.3% | 84.7% | 71.7% | 46.6% | 100.0% |  |
|  | **For-Profit** | 24.0% (23.9-24.2) | 44.1% | 17.5% | 0.6% | 0 | 39.9% | 0 |  |
| **Hospital Type** | **General Medical/Surgical** | 93.6% (93.5-93.6) | 90.7% | 94.0% | 97.2% | 97.6% | 87.3% | 100% | <0.001 |
|  | **Other** | 6.4% (6.4-6.5) | 9.3% | 6.0% | 2.8% | 2.4% | 12.8% | 0 |  |
| **Urban/Rural** | **Metro** | 96.4% (96.3-96.4) | 98.6% | 88.8% | 98.4% | 96.4% | 94.2% | 47.2% | <0.001 |
|  | **Micro** | 2.6% (2.6-2.7) | 1.1% | 7.5% | 1.3% | 3.0% | 3.1% | 31.5% |  |
|  | **Rural** | 1.0% (1.0-1.0) | 0.34% | 3.8% | 0.4% | 0.6% | 2.7% | 21.3% |  |
| **Racial/Ethnic Makeup** | **Percent White** | 58.6% (58.5, 58.7) | 64.0% | 52.1% | 51.5% | 49.1% | 87.1% | 95.7% | <0.001 |

**Appendix 3: Distribution of Hospital Characteristics of Readmission Hospitals in Dyads**

| **Variable** | | **All States (N=596,772)**  **(%, 95% CI)** | **Florida (N=278,345)** | **Georgia (n=84,265)** | **Maryland (n=54,156)** | **New York (n=164,501)** | **Utah (n=14,323)** | **Vermont (n=1,182)** | **p** |
| --- | --- | --- | --- | --- | --- | --- | --- | --- | --- |
| **Bedsize** | **<100 Beds** | 11.1% (11.0-11.2) | 14.0% | 16.2% | 10.3% | 2.9% | 20.8% | 33.6% | <0.001 |
|  | **100-199 Beds** | 16.7% (16.6-16.8) | 17.5% | 15.7% | 19.5% | 14.0% | 26.8% | 10.7% |  |
|  | **200-299 Beds** | 17.0% (16.9-17.0) | 16.0% | 17.1% | 24.5% | 16.0% | 17.2% | 13.1% |  |
|  | **300-399 Beds** | 13.6% (13.5-13.6) | 15.7% | 10.5% | 15.8% | 10.8% | 13.1% | 0 |  |
|  | **400-499 Beds** | 10.6% (10.5-10.7) | 9.4% | 10.9% | 14.9% | 11.9% | 0 | 42.6% |  |
|  | **≥500 Beds** | 31.1% (31.0-31.2) | 27.4% | 29.6% | 15.0% | 44.5% | 22.1% | 0 |  |
| **Teaching** | **Non-Teaching** | 27.7% (27.6-27.8) | 36.4% | 37.5% | 18.6% | 10.0% | 38.1% | 44.3% | <0.001 |
|  | **Teaching** | 72.3% (72.2-72.4) | 63.6% | 62.6% | 81.4% | 90.0% | 61.9% | 55.7% |  |
| **Ownership** | **Government** | 12.2% (12.1-12.2) | 12.8% | 4.5% | 0 | 18.9% | 15.0% | 0 | <0.001 |
|  | **Church** | 6.7% (6.7-6.8) | 4.3% | 4.7% | 13.5% | 10.4% | 0 | 0 |  |
|  | **Other Not-for-profit** | 56.2% (56.0-56.3) | 37.7% | 71.5% | 84.8% | 70.8% | 45.2% | 100% |  |
|  | **For-Profit** | 25.0% (24.9-25.1) | 45.3% | 19.3% | 1.7% | 0 | 39.8% | 0 |  |
| **Hospital Type** | **General Medical/Surgical** | 89.0% (89.0-89.1) | 85.0% | 90.1% | 92.2% | 95.0% | 80.7% | 100% | <0.001 |
|  | **Other** | 11.0% (10.9-11.0) | 15.0% | 9.9% | 7.8% | 5.0% | 19.3% | 0 |  |
| **Urban/Rural** | **Metro** | 96.6% (96.6-96.7) | 98.8% | 89.4% | 98.4% | 96.5% | 95.5% | 51.5% | <0.001 |
|  | **Micro** | 2.5% (2.4-2.5) | 0.9% | 7.0% | 1.3% | 2.9% | 2.3% | 29.5% |  |
|  | **Rural** | 0.9% (0.9-1.0) | 0.3% | 3.7% | 0.3% | 0.6% | 2.3% | 19.0% |  |
| **Racial/Ethnic Makeup: Percent White** |  | 59.1% (59.0, 59.2) | 62.9% | 55.6% | 49.3% | 51.9% | 87.1% | 98.9% | <0.001 |

**Appendix 4: Sensitivity Analyses for Additional Hospital/Payer Characteristics, All Readmissions**

| Model | All* | Florida | Georgia | Maryland | New York | Utah | Vermont |
| --- | --- | --- | --- | --- | --- | --- | --- |
| All Covariates-Same System Only | -3.61 (-3.70, -3.52) | -3.55 (-3.69, -3.42) | -3.17 (-3.28, -3.07) | -13.11 (-13.50, -12.72) | -12.69 (-13.09, -12.30) | -0.44 (-0.48, -0.40) | --ǂ |
| All covariates-Different Systems | -2.84 (-2.87, -2.81) | -3.38 (-3.43, -3.34) | -2.21 (-2.23, -2.18) | -4.04 (-4.13, -3.95) | -2.58 (-2.63, -2.54) | -0.39 (-0.42, -0.37) | -0.63 (-0.72, -0.55) |
| All Covariates + Trauma Hospital Status | -3.04 (03.07, -3.01) | -3.52 (-3.56, -3.48) | -2.36 (-2.39, -2.33) | -5.29 (-5.40, -5.19) | -3.11 (-3.16, -3.06) | -0.38 (0.40, -0.36) | -0.63 (-0.72, -0.55) |
| All Covariates + Critical Access Hospital Status | -3.03 (-3.06, -3.01) | -3.51 (-3.55, -3.47) | -2.36 (-2.39, -2.34) | -4.98 (-5.08, -4.87) | -3.09 (-3.14, -3.04) | -0.37 (-0.39, -0.35) | -0.64 (-0.71, -0.56) |
| All Covariates + Accountable care organization | -3.03 (-3.06, -3.01) | -3.51 (-3.55, -3.47) | -2.36 (-2.39, -2.33) | -5.00 (-5.10, -4.90) | -3.10 (-3.15, -3.05) | -0.39 (-0.41, -0.37) | -0.63 (-0.71, -0.54) |
| All Covariates + Insurances | -3.09 (-3.11, -3.06) | -3.52 (-3.56, -3.47) | -2.36 (-2.39, -2.33) | -5.02 (-5.12, -4.92) | -3.18 (-3.23, -3.13) | -0.37 (-0.39, -0.35) | -0.63 (-0.72, -0.54) |

*Dummy variables for states were used in the multi-state analysis.

-ǂ Vermont did not have any hospitals identified as in the same system based on the AHA survey.

All: Modeling the relationship between index and readmission hospital and volume of shared patients adjusted for hospital characteristics (bed size, teaching status, hospital ownership, and type of hospital), percent of white individuals in the zip code tabulation area of the patient’s home zip code, and HHI (75% of market)

Same system: Hospitals are part of the same hospital/healthcare system, identified by both hospitals having the same “sysid” on the AHA survey

Different system: Hospitals are part of different hospital/healthcare systems, identified by both hospitals having different “sysid” on the AHA survey

Trauma Hospital: Admission and/or readmission hospital trauma center designation (measured for admission-readmission pair)

Critical Access Hospital: Admission and/or readmission hospital critical access hospital designation (measured for admission-readmission pair)

Accountable Care Organization: Admission and/or readmission hospital is a member of an accountable care organization (measured for admission-readmission pair)

Insurances offered: Admission and/or readmission hospital offers health insurance (Medicare advantage, Medicaid Managed Care, marketplace, small or large group plan, other) (measured for admission-readmission pair)

**Appendix 5: Distribution of Hospital Characteristics of Index Hospitals in Dyads, 30-day readmissions**

| Variable |  | All States  (309640) (%, 95% CI) | Florida  (151610) | Georgia  (42155) | Maryland  (26830) | New York  (81141) | Utah  (7350) | Vermont  (554) | p |
| --- | --- | --- | --- | --- | --- | --- | --- | --- | --- |
| Bedsize | <100 Beds | 8.6% (8.5-8.7) | 9.9% | 13.0% | 7.1% | 3.1% | 20.3% | 42.4% | <0.001 |
|  | 100-199 Beds | 16.0% (15.9, 16.1) | 18.2 | 14.9% | 19.1% | 10.6% | 26.8% | 7.8% |  |
|  | 200-299 Beds | 18.4% (18.3, 18.5) | 18.0% | 16.7% | 27.8% | 17.1% | 16.9% | 13.2% |  |
|  | 300-399 Beds | 14.9% (14.8, 15.1) | 17.1% | 15.6% | 17.9% | 119% | 13.7% | 0 |  |
|  | 400-499 Beds | 10.7% (10.6, 10.8) | 9.9% | 12.1% | 13.4% | 11.5% | 0 | 36.6% |  |
|  | ≥500 Beds | 31.3% (31.2, 31.5) | 26.9% | 31.8% | 14.9% | 45.9% | 22.3% | 0 |  |
| Teaching | Non-Teaching | 26.8% (26.6, 27.0) | 34.7% | 35.8% | 14.0% | 10.5% | 37.2% | 50.2% | <0.001 |
|  | Teaching | 73.2% (73.1, 73.4) | 65.3% | 64.2% | 86.0% | 89.5% | 62.8% | 49.8% |  |
| Ownership | Government | 12.6% (12.4, 12.7) | 13.4% | 4.1% | 0 | 19.5% | 14.1% | 0 | <0.001 |
|  | Church | 6.3% (6.2, 6.3) | 4.1% | 4.3% | 14.6% | 9.1% | 0 | 0 |  |
|  | Other Not-for-profit | 56.1% (56.0, 56.3) | 38.0% | 74.7% | 85.1% | 71.4% | 48.5% | 100.0% |  |
|  | For-Profit | 25.1% (24.9, 25.2) | 44.5% | 16.9% | 0.3% | 0 | 39.5% | 0 |  |
| Hospital Type | General Medical/Surgical | 93.6% (93.5, 93.7) | 90.8% | 94.4% | 97.9% | 97.4% | 87.9% | 100% | <0.001 |
|  | Other | 6.4% (6.3, 6.5) | 9.2% | 5.6% | 2.1% | 2.6% | 12.2% | 0 |  |
| Urban/Rural | Metro | 96.7% (96.7, 96.8) | 98.7% | 89.4% | 98.6% | 96.7% | 93.9% | 50.2% | <0.001 |
|  | Micro | 2.4% (2.3, 2.4) | 1.0% | 7.1% | 1.1% | 2.7% | 3.1% | 27.4% |  |
|  | Rural | 0.9% (0.9, 0.9) | 0.3% | 3.4% | 0.3% | 0.5% | 3.0% | 22.4% |  |
| Racial/Ethnic Makeup* (% White) |  | 58.0% (57.9, 58.1) | 64.0% | 52.1% | 51.5% | 49.1% | 87.0% | 95.7% | <0.001 |

*Note 2.5% of data are missing.

**Appendix 6: Distribution of Hospital Characteristics of Readmission Hospitals in Dyads, 30-day readmissions**

| Variable |  | All States  n=390640  (%, 95% CI) | Florida  N=151610 | Georgia  N=42155 | Maryland  N=26830 | New York  N=81141 | Utah  N=7350 | Vermont  N=554 | p |
| --- | --- | --- | --- | --- | --- | --- | --- | --- | --- |
| Bedsize | <100 Beds | 15.1% (15.0, 15.2) | 19.7% | 21.3% | 2.7% | 24.0% | 31.2% | 33.6% | <0.001 |
|  | 100-199 Beds | 17.2% (17.1, 17.4) | 17.4% | 15.5% | 19.5% | 16.0% | 29.2% | 10.6% |  |
|  | 200-299 Beds | 15.6% (15.5, 15.8) | 14.0% | 15.8% | 22.5% | 16.3% | 15.4% | 12.8% |  |
|  | 300-399 Beds | 12.6% (12.5, 12.7) | 14.3% | 9.5% | 14.1% | 10.5% | 12.2% | 0 |  |
|  | 400-499 Beds | 10.0% (10.0, 10.1) | 8.6% | 10.5% | 15.3% | 11.2% | 0 | 44.8% |  |
|  | ≥500 Beds | 29.5% (29.3, 29.6) | 25.9% | 27.4% | 14.5% | 43.2% | 19.2% | 0 |  |
| Teaching | Non-Teaching | 30.3% (30.2, 30.5) | 39.9% | 38.7% | 22.3% | 9.5% | 43.5% | 42.4% | <0.001 |
|  | Teaching | 69.7% (69.5, 69.8) | 60.1% | 61.3% | 77.7% | 90.5% | 56.5% | 57.6% |  |
| Ownership | Government | 12.0% (11.9, 12.1) | 12.0% | 4.3% | 0 | 19.8% | 15.0% | 0 | <0.001 |
|  | Church | 6.4% (6.4, 6.5) | 3.9% | 4.6% | 12.3% | 10.9% | 0 | 0 |  |
|  | Other Not-for-profit | 53.2% (53.0, 53.4) | 35.1% | 69.2% | 84.3% | 69.3% | 40.9% | 100% |  |
|  | For-Profit | 28.3% (28.2, 28.5) | 49.0% | 22.0% | 3.4% | 0 | 44.1% | 0 |  |
| Hospital Type | General Medical/Surgical | 82.3% (82.2, 82.4) | 76.7% | 84.0% | 85.1% | 92.0% | 71.0% | 100% | <0.001 |
|  | Other | 17.7% (17.5, 17.8) | 23.3% | 16.0% | 14.9% | 8.0% | 29.0% | 0 |  |
| Urban/Rural | Metro | 97.1% (97.1, 97.2) | 99.1% | 90.7% | 98.6% | 96.9% | 95.7% | 54.0% | <0.001 |
|  | Micro | 2.1% (2.0, 2.1) | 0.7% | 6.0% | 1.2% | 2.7% | 2.0% | 28.0% |  |
|  | Rural | 0.8% (0.8, 0.8) | 0.2% | 3.3% | 0.3% | 0.5% | 2.3% | 18.1% |  |
| Racial/Ethnic Makeup* (% White) |  | 58.5% (58.4, 58.6) | 62.8% | 55.6% | 49.3% | 51.9% | 87.2% | 98.9% | <0.001 |

*Note 2.5% of data are missing.

**Appendix 7: Linear Regression Models, 30-day readmissions**

| Model | All* | Florida | Georgia | Maryland | New York | Utah | Vermont |
| --- | --- | --- | --- | --- | --- | --- | --- |
| Unadjusted | -1.8 (-1.8, -1.8) | -2.0 (-2.0, -1.9) | -1.3 (-1.3, -1.3) | -3.0 (-3.1, -2.9) | -1.7 (01.7, -1.6) | -0.4 -0.4, -0.4) | -0.7 (-0.8, -0.5) |
| Hospital Characteristics | -1.7 (-1.7, -1.6) | -1.9 (-2.0, -1.9) | -1.2 (01.2, -1.2) | -3.0 (-3.0, -2.9) | -1.5 (-1.6, -1.5) | -0.4 (-0.4, -0.3) | -0.6 (-0.7, -0.5) |
| Hospital + Patient Characteristics | -1.8 (-1.8, -1.7) | -2.0 (-2.0, -1.9) | -1.2 (-1.2, -1.2) | -3.2 (-3.3, -3.1) | -1.9 (-1.9, -1.8) | -0.4 (-0.4, -0.3) | -0.9 (-0.9, -0.8) |
| Market | -1.8 (-1.8, -1.8) | -2.0 (-2.0, -2.0) | -1.3 (-1.3, -1.2) | -3.0 (-3.1, -2.9) | -1.6 (-1.7, 01.6) | -0.3 (-0.3, -0.3) | -0.7 (-0.8, -0.6) |
| All | -1.8 (-1.8, -1.8) | -2.0 (-2.0, -1.9) | -1.2 (-1.2, -1.2) | -3.3 (-3.3, -3.2) | -1.9 (-1.9, -1.8) | -0.3 (-0.4, -0.3) | -0.8 (-0.9, -0.8) |

*Dummy variables for states were used in the multi-state analysis.

Unadjusted: Modeling the relationship between index and readmission hospital and volume of shared patients.

Hospital Characteristics: bed size, teaching status, hospital ownership, and type of hospital—note each covariate is the characteristics of the dyad (ex. index-teaching, readmit-nonteaching)

Hospital and Patient Characteristics: Above plus percent of white patients in the zip code tabulation area of the patient’s home zip code.

Market: HHI (75% of market)

All: Hospital, patient, market as above.

**Appendix 8: Distribution of Hospital Characteristics of Index Hospitals in Dyads, First Admission-Readmission Pair Only**

| Variable |  | All States  (N=395,318) (%, 95% CI) | Florida  (N=176,966) | Georgia  (N=58,578) | Maryland  (N=36,764) | New York  (N=111,667) | Utah  (N=10,376) | Vermont  (N=967) | p |
| --- | --- | --- | --- | --- | --- | --- | --- | --- | --- |
| Bedsize | <100 Beds | 8.1% (8.0, 8.2) | 9.3% | 11.9% | 6.2% | 3.5% | 19.5% | 45.1% | <0.001 |
|  | 100-199 Beds | 16.5% (16.4, 16.6) | 18.0% | 16.8% | 20.2% | 11.8% | 26.6% | 11.0% |  |
|  | 200-299 Beds | 19.1% (19.0, 19.2) | 19.3% | 18.2% | 29.0% | 16.1% | 18.6% | 14.9% |  |
|  | 300-399 Beds | 14.2% (14.0, 14.3) | 17.1% | 11.3% | 18.3% | 9.8% | 14.5% | 0 |  |
|  | 400-499 Beds | 9.6% (10.5, 10.7) | 9.6% | 10.6% | 12.4% | 12.4% | 0 | 29.1% |  |
|  | ≥500 Beds | 31.6% (31.5, 31.7) | 26.8% | 31.2% | 14.1% | 46.5% | 20.8% | 0 |  |
| Teaching | Non-Teaching | 26.9% (26.8, 27.1) | 35.3% | 37.2% | 14.9% | 11.2% | 36.6% | 56.1% | <0.001 |
|  | Teaching | 73.1% (72.9, 73.2) | 64.7% | 62.8% | 85.1% | 88.8% | 63.4% | 44.0% |  |
| Ownership | Government | 12.1% (12.0, 12.2) | 13.1% | 4.8% | 0 | 18.2% | 14.3% | 0 | <0.001 |
|  | Church | 7.2% (7.1, 7.3) | 4.6% | 5.1% | 14.8% | 10.6% | 0 | 0 |  |
|  | Other Not-for-profit | 57.5% (57.4, 57.7) | 38.5% | 73.2% | 84.9% | 71.2% | 47.8% | 100.0% |  |
|  | For-Profit | 23.2% (23.0, 23.3) | 43.9% | 16.9% | 0.3% | 0 | 37.9% | 0 |  |
| Hospital Type | General Medical/Surgical | 94.9% (94.8, 94.9) | 92.6% | 95.8% | 98.7% | 97.3% | 88.7% | 100% | <0.001 |
|  | Other | 5.2% (5.1, 5.2) | 7.4% | 4.3% | 1.3% | 2.8% | 11.3% | 0 |  |
| Urban/Rural | Metro | 95.9%% (95.9, 96.0) | 98.7% | 89.4% | 98.6% | 96.7% | 93.9% | 50.2% | <0.001 |
|  | Micro | 2.9% (2.9, 3.0) | 1.0% | 7.1% | 1.1% | 2.7% | 3.1% | 27.4% |  |
|  | Rural | 1.1% (1.1, 1.2) | 0.3% | 3.4% | 0.3% | 0.5% | 3.0% | 22.4% |  |
| Racial/Ethnic Makeup* | Lower percentage of white people than the mean across all datasets | 52.1% (52.0, 52.3) | 42.3% | 69.2% | 72.9% | 57.2% | -- | 0 | <0.001 |
|  | Higher percentage of white people than the mean across all datasets | 45.1%% (44.9, 45.2) | 57.7% | 30.8% | 27.1% | 42.2% | -- | 100% |  |

*Note 2.8% of data are missing.

**Appendix 9: Distribution of Hospital Characteristics of Readmission Hospitals in Dyads, First Admission-Readmission Pair Only**

| Variable |  | All States  (N=395,318) (%, 95% CI) | Florida  (N=176,966) | Georgia  (N=58,578) | Maryland  (N=36,764) | New York  (N=111,667) | Utah  (N=10,376) | Vermont  (N=967) | p |
| --- | --- | --- | --- | --- | --- | --- | --- | --- | --- |
| Bedsize | <100 Beds | 11.5% (11.4, 11.6) | 14.2% | 16.9% | 12.3% | 3.2% | 21.4% | 28.0% | <0.001 |
|  | 100-199 Beds | 16.4% (16.3, 16.5) | 17.2% | 14.8% | 17.5% | 14.8% | 26.2% | 9.7% |  |
|  | 200-299 Beds | 16.4% (16.2, 16.5) | 14.8% | 16.7% | 22.9% | 16.5% | 16.4% | 12.0% |  |
|  | 300-399 Beds | 13.7% (13.6, 13.8) | 16.5% | 11.0% | 14.7% | 10.5% | 12.4% | 0 |  |
|  | 400-499 Beds | 11.4% (11.3, 11.5) | 9.8% | 11.5% | 18.3% | 12.2% | 0 | 50.3% |  |
|  | ≥500 Beds | 30.7% (30.5, 30.8) | 27.5% | 29.2% | 14.2% | 42.7% | 26.7% | 0 |  |
| Teaching | Non-Teaching | 28.1% (29.7, 28.2 | 36.9% | 37.4% | 20.5% | 10.8% | 37.9% | 37.8% | <0.001 |
|  | Teaching | 71.9% (71.8, 72.1) | 63.1% | 62.6% | 79.5% | 89.2% | 62.1% | 62.3% |  |
| Ownership | Government | 11.6% (11.5, 11.7) | 13.6% | 4.7% | 0 | 15.9% | 13.3% | 0 | <0.001 |
|  | Church | 6.9% (6.9, 7.0) | 4.7% | 4.6% | 12.8% | 10.4% | 0 | 0 |  |
|  | Other Not-for-profit | 58.4% (58.3, 58.6) | 39.5% | 71.3% | 84.9% | 73.7% | 47.7% | 100% |  |
|  | For-Profit | 23.0% (22.9, 23.2) | 42.2% | 19.5% | 2.3% | 0 | 39.1% | 0 |  |
| Hospital Type | General Medical/Surgical | 88.9% (88.8, 89.0) | 85.4% | 89.0% | 89.6% | 94.4% | 83.6% | 100% | <0.001 |
|  | Other | 11.2% (11.1, 11.2) | 14.6% | 11.0% | 10.4% | 5.6% | 16.4% | 0 |  |
| Urban/Rural | Metro | 96.4% (96.4, 96.5) | 98.8% | 89.6% | 98.4% | 96.0% | 95.2% | 56.9% | <0.001 |
|  | Micro | 2.6% (2.6, 2.7) | 0.8% | 6.7% | 1.3% | 3.5% | 2.6% | 26.5% |  |
|  | Rural | 1.0% (0.9, 1.0) | 0.3% | 3.7% | 0.3% | 0.5% | 2.3% | 16.7% |  |
| Racial/Ethnic Makeup* | Lower percentage of white people than the mean across all datasets | 51.8% (51.7, 52.0) | 41.0% | 69.8% | 77.0% | 56.5% | -- | 0 | <0.001 |
|  | Higher percentage of white people than the mean across all datasets | 45.5% (45.3, 45.6) | 59.0% | 30.1% | 23.0% | 43.1% | -- | 100% |  |

*Note 2.8% of data are missing.

**Appendix 10: Linear Regression Models, First Admission-Readmission Pair Only**

| Model | All* | Florida | Georgia | Maryland | New York | Utah | Vermont |
| --- | --- | --- | --- | --- | --- | --- | --- |
| Unadjusted | -1.6 (-1.7, -1.6) | -2.0 (-2.0, -1.9) | -1.3 (-1.3, -1.3) | -3.2 (-3.2, -3.0) | -1.6 (-1.7, -1.6) | -0.4 (-0.4, -0.4) | -0.6 (-0.8, -0.5) |
| Hospital Characteristics | -1.5 (-1.6, -1.5) | -1.9 (-2.0, -1.9) | -1.2 (-1.2, -1.2) | -3.1 (-3.2, -3.0) | -1.5 (-1.5, -1.4) | -0.3 (-0.4, -0.3) | -0.6 (-0.7, -0.5) |
| Hospital + Patient Characteristics | -1.6 (-1.7, -1.6) | -2.0 (-2.0, -1.9) | -1.2 (-1.3, -1.2) | -3.3 (-3.4, -3.2) | -1.8 (-1.8, -1.7) | -0.4 (-0.4, -0.3) | -0.9 (-0.9, -0.8) |
| Market | -1.7 (-1.7, -1.7) | -2.0 (-2.0, -1.9) | -1.3 (-1.3, -1.3) | -3.2 (-3.3, -3.1) | -1.6 (-1.6, -1.6) | -0.3 (-0.3, -0.3) | -0.7 (-0.8, -0.6) |
| All | -1.7 (-1.7, -1.7) | -2.0 (-2.0, -1.9) | -1.2 (-1.3, -1.2) | -3.4 (-3.5, -3.3) | -1.8 (-1.8, -1.7) | -0.34 (-0.4, -0.3) | -0.8 (-0.9, -0.7) |

*Dummy variables for states were used in the multi-state analysis.

Unadjusted: Modeling the relationship between index and readmission hospital and volume of shared patients.

Hospital Characteristics: bed size, teaching status, hospital ownership, and type of hospital—note each covariate is the characteristics of the dyad (ex. index-teaching, readmit-nonteaching)

Hospital and Patient Characteristics: Above plus percent of white patients in the zip code tabulation area of the patient’s home zip code.

Market: HHI (75% of market)

All: Hospital, patient, market as above.

**Appendix 11: Linear Regression Models, Outcome is Percent of Total Admissions to Admission and Readmission Hospital**

| Model | All* | Florida | Georgia | Maryland | New York | Utah | Vermont |
| --- | --- | --- | --- | --- | --- | --- | --- |
| Unadjusted | -0.024 (-0.024, -0.023) | -0.025 (-0.026, -0.025) | -0.024 (-0.025, -0.024) | -0.055 (-0.057, -0.053) | -0.021 (-0.022, -0.021) | -0.01 (-0.01, -0.01) | -0.026 (-0.029, -0.023) |
| Hospital Characteristics | -0.025 (-0.025, -0.024) | -0.025 (-0.026, -0.025) | -0.025 (-0.025, -0.024) | -0.057 (-0.058, -0.055) | -0.025 (-0.026, -0.025) | -0.015 (-0.015, -0.014) | -0.026 (-0.028, -0.023) |
| Hospital + Patient Characteristics | -0.019 (-0.019, -0.019) | -0.019 (-0.019, -0.019) | -0.020 (-0.021, -0.024) | -0.074 (-0.075, -0.073) | -0.015 (-0.015, -0.015) | -0.016 (-0.017, -0.015) | -0.025 (-0.027, -0.023) |
| Market | -0.019 (-0.019, -0.019) | -0.020 (-0.020, -0.020) | -0.020 (-0.021, -0.020) | -0.046 (-0.047, -0.045) | -0.014 (-0.014, -0.014) | -0.016 (-0.017, -0.015) | -0.026 (-0.028, -0.023) |
| All | -0.020 (-0.020, -0.019) | -0.020 (-0.020, -0.019) | -0.021 (-0.021, -0.021) | -0.076 (-0.077, -0.075) | -0.015 (-0.015, -0.015) | -0.017 (-0.018, -0.016) | -0.025 (-0.027, -0.023) |

*Dummy variables for states were used in the multi-state analysis.

Unadjusted: Modeling the relationship between index and readmission hospital and volume of shared patients.

Hospital Characteristics: bed size, teaching status, hospital ownership, and type of hospital—note each covariate is the characteristics of the dyad (ex. index-teaching, readmit-nonteaching)

Hospital and Patient Characteristics: Above plus percent of white patients in the zip code tabulation area of the patient’s home zip code.

Market: HHI (75% of market)

All: Hospital, patient, market as above.
